# Variability in the temporal dynamics of object-based attentional selection

**DOI:** 10.1371/journal.pone.0294252

**Published:** 2023-11-17

**Authors:** Hao Lou, Monicque M. Lorist, Karin S. Pilz

**Affiliations:** 1 Department of Experimental Psychology, University of Groningen, Groningen, The Netherlands; 2 Department of Biomedical Sciences of Cells and Systems, Cognitive Neuroscience Center, University of Groningen, Groningen, The Netherlands; 3 Cito Institute for Test Development, Arnhem, The Netherlands; Institute of Psychology Chinese Academy of Sciences, CHINA

## Abstract

Our attention can be directed to specific locations in our visual field (space-based attention), or to specific objects (object-based attention). However, object-based attention tends to be less pronounced than space-based attention and can vary greatly between individuals. Here we investigated whether the low prevalence of object-based effects is related to variability in the temporal dynamics of attentional selection. We manipulated cue-to-target intervals from 50 to 600 ms in a two-rectangle discrimination task. Space- and object-based effects were measured at the group level and for individual participants. We used bootstrapping to highlight cue-to-target intervals with maximal space- and object-based effects, and fast Fourier transform (FFT) to investigate rhythmic sampling of locations within and between objects. Whereas overall, space-based effects were robust and stable across all cue-to-target intervals for most participants, object-based effects were small and were only found for a small subset of participants in the different cue-to-target intervals. In the frequency domain, only a small number of participants exhibited significant periodicities, prompting the need for further investigation and consideration. Overall, our study suggests variability in the temporal dynamics of object-based effects underlying their low prevalence, a finding that needs to be further investigated in future studies.

## 1. Introduction

When we open our eyes, a manifold of visual information enters simultaneously. There is no limit to the amount of incoming information at this early stage of visual processing. However, the brain does not possess an unlimited capacity to process it all at the same time, and different types of attention aid the selection of relevant information. For example, attention can be allocated to a spatial location in the visual field [1]. This so-called *space-based attention* improves the processing of information in the attended region. *Object-based attention*, in comparison, directs attention to specific objects in visual space. Parts within an object are considered to be processed more quickly and accurately than parts across different objects for object-based attention [2–5].

In a commonly used paradigm to study space- and object-based effects, two rectangular objects are presented horizontally or vertically on the screen. A cue appears at one end of one rectangle, shortly followed by a target. The target can appear at the cued location (*valid trials*) or uncued locations, either within the cued rectangle (*invalid same-object trials*) or in the uncued rectangle (*invalid different-object trials*). Participants are asked to detect or discriminate the target as quickly as possible. Most importantly, the cue is equidistant from the target on both types of invalid trials. Interestingly, however, object-specific advantages are commonly shown by faster responses on the same- compared to different-object trials, which indicates that attention spreads along the object. This paradigm, and modified versions of it, have been widely used to study space- and object-based attentional selection [4–10].

In general, object-based effects have been found to be smaller and not as robust as space-based effects [8, 9, 11, 12], and are affected by changes to the paradigm, including the probability of targets appearing at the cued location [6, 8, 9, 13] and the duration of the interval between the cue and the target [6, 14, 15]. Testing 240 participants, Pilz et al. [10] investigated the strength of space- and object-based effects on the level of individual participants. Overall, they observed large variability for both space- and object-based effects. But whereas space-based effects were large and were found in nearly all participants across three conventional paradigms [4, 5, 16], object-based effects were small and only present in a minority of participants.

Despite the established knowledge about object-based attention in previous studies, there is limited understanding of how this attentional effect unfolds over time and whether its temporal emergence varies across individuals. Several studies have found that the length of cue-to-target intervals modulates object-based attention such that the magnitude of object-based effects declines with an increasing cue-to-target interval [6, 14, 15]. More recently, Helfrich et al. [17] showed that object-based attention fluctuates over periods of 500–1700 ms in behavioral performance and that this fluctuation is shaped by neural oscillations. Also, other attentional processes have been found to [17, 18], in particular, in the temporal domain related to the attentional blink [19–21]. Therefore, it is reasonable to assume that the cue-to-target interval at which maximal object-based effects occurs, is not consistent across individuals. To test our hypothesis, we adopted the commonly used two-rectangle discrimination task described above [8, 10]. Instead of a consistent cue-to-target interval of 200 ms, we systematically changed intervals from 50 ms to 600 ms to assess object-based effects over time.

Our hypothesis ties in with research that suggests visual attention to be a rhythmic rather than a continuous process. Attention samples the environment in a particular frequency that leads to an either enhanced or diminished perceptual sensitivity of location within the visual field [22–25]. Attentional rhythms in the theta (4–8 Hz) frequency range have been found in different studies related to attentional cueing, stimulus detection, and visual search [22, 25–31]. Fiebelkorn et al. [29] investigated rhythmic sampling of attentional selection in behavior and found a 4 Hz rhythmic pattern in participants’ sampling locations between objects (object-based effects). However, it was observed that the antiphase relationship characterizing this rhythmic pattern exhibited substantial variability across individuals (as depicted in Fig 3C). This observation led us to question whether the periodic pattern of object-based effects may differ between individuals, potentially accounting for the individual variability observed in the object-based effects.

Therefore, in the current study, we investigated individual variability in the temporal dynamics of space- and object-based attention using analyses in the time- and frequency domain. In the time domain, we examined the occurrence and magnitude of space- and object-based effects on both the group and individual levels. In the frequency domain, we focused on potential periodic patterns associated with space- and object-based effects and examined whether these patterns exhibited variability across individuals. Given that object-based effects commonly occur at intervals between 100 ms and 400 ms, we decided to test performance for cue-to-target intervals between 50 ms and 600 ms, rather than between 300 ms and 1000 ms, as done in previous studies [22, 28, 32, 33]. In the time domain, we first assessed overall object-based effects across time using parametric statistical methods (i.e., ANOVA and t-tests). We then used a non-parametric bootstrapping method, similar to Pilz et al. [10], to assess the temporal dynamics of space- and object-based effects for each participant. In the frequency domain, rhythmic patterns of space- and object-based attention were measured by assessing the periodicities of attended locations using the Fast Fourier Transform (FFT), using the methods described by Fiebelkorn et al. [29].

## 2. Methods

### 2.1 Participants

In previous research examining object-based effects, sample sizes have commonly ranged from 15 to 20 participants (e.g., [4, 5, 34–36]). Given our main interest in the between-subject variability of the effects, we opted for a larger sample size. Participants were recruited from the University of Groningen in exchange for course credits. Four participants were excluded from the analysis: two participants quit during the experiment, one participant pressed repetitive keys for more than half of the trials, and one participant had a visual acuity below 0.8 (measured by the Early Treatment Diabetic Retinopathy Study logarithmic vision chart; [37]). The final sample comprised data from forty-three participants (18–27 years, *M* = 19.9; *SD* = 1.7, 12 males) with normal or corrected-to-normal visual acuity. The Ethical Committee Psychology of the University of Groningen approved the experiment and procedures. All participants gave written informed consent, and the experiment was conducted in accordance with the Declaration of Helsinki (2008). The research data were obtained and stored without identifiers that can be directly linked back to the participants.

This study was preregistered on the Open Science Framework (OSF) before commencing data analysis. A detailed pre-registration can be found at https://osf.io/5z7wn. In order to evaluate the statistical power of our study, we conducted a post-hoc power analysis using G*Power. We employed the observed effect size of Cohen’s d = -0.85, set the significance level (alpha) at α = 0.05, and used a sample size of n = 43. The power analysis yielded a statistical power of .999, indicating a high likelihood of detecting object-based effects in our study.

### 2.2 Apparatus, stimuli, and procedure

All stimuli and experimental trials were created using MATLAB 9.5 (The MathWorks) and the Psychophysics toolbox version 3.0.16 [38, 39] running under Windows 10 and displayed on a 1920 × 1080 monitor with a refresh rate of 100 Hz. Participants viewed the stimuli binocularly at a distance of 57 cm while seated in an adjustable chair in a darkened room.

**Fig 1:**
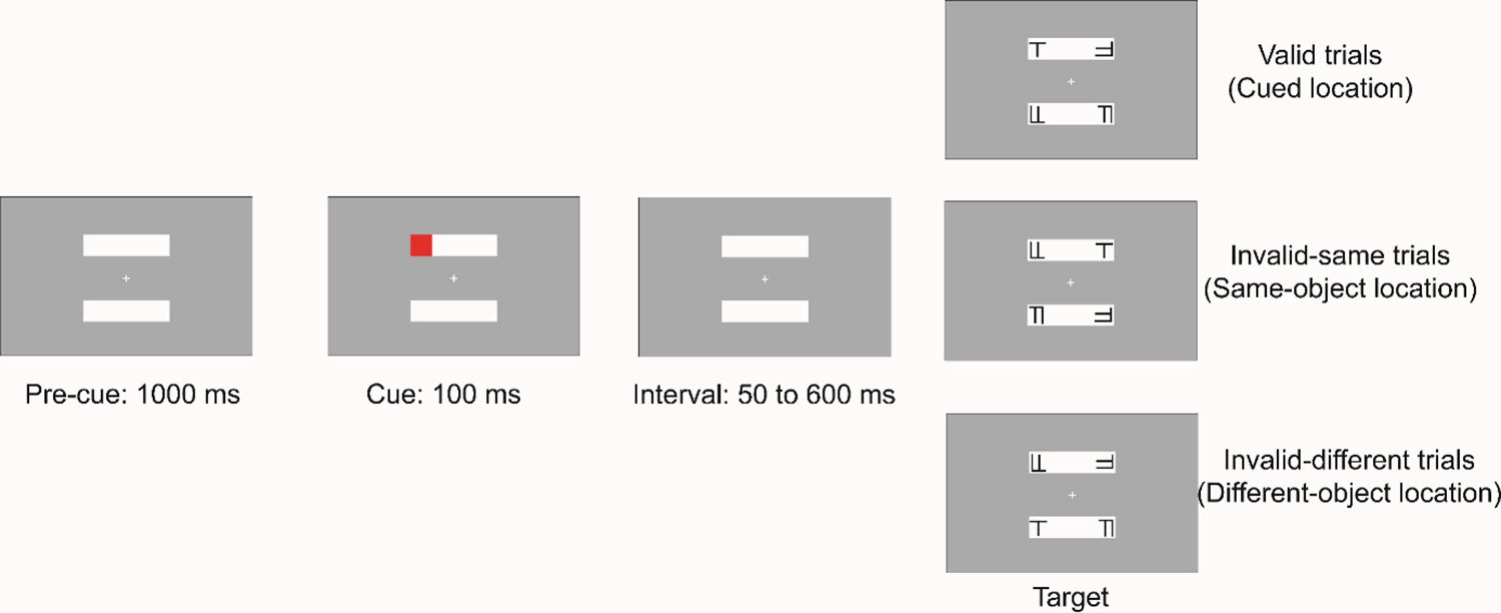
Schematic diagram of one trial sequence for each of the three conditions.

Stimuli and procedure were similar as in the discrimination task from Pilz et al.[10] and Lou et al. [8]. The stimulus display was composed of two white rectangles (each 1.6 × 8.2 degrees visual angle) and a white fixation cross (0.45 × 0.45 degrees visual angle) located in the center of the screen. The background of the display was grey (Fig 1). The two rectangles were oriented horizontally and located above and below the fixation cross. Object-based effects were found to be more prominent for horizontal objects [7, 10, 40, 41]. Given the emphasis on temporal aspects of object-based attention and the duration of the experiment, only horizontal objects were included in the current study.

At the start of each trial, two rectangles were presented for 1000 ms on the screen. A red square cue (1.6 x 1.6 degrees visual angle) was then presented for 100 ms in one of the four corners of the two rectangles. Following a randomly sampled 50–600 ms cue-to-target interval (in steps of 10 ms, corresponding to a sampling frequency of 100 Hz), one target and three distractors appeared. The target was presented in one of three locations: the cued location (*valid trials*), the opposite end of the cued rectangle (*invalid-same trials*), or the same end of the uncued rectangle (*invalid-different trials*). Targets were either a letter T or a letter L, presented with equal probability in an upright orientation, or rotated 90, 180, or 270 degrees. Distractor items were T-L hybrid characters, which had the same size as the target items and were also presented in one of the four orientations (0, 90, 180, and 270 degrees). Participants were asked to identify whether the target was a “T” or an “L” by pressing the ‘‘/”key (for T’s) or the ‘‘z” key (for L’s) on a QWERTY keyboard as quickly and accurately as possible. The target and distractors stayed on the screen until the participant made a response. Each participant completed 32 blocks with 50 trials, 1600 trials in total, of which 80% were valid trials, 10% were invalid-same trials, and 10% were invalid-different trials. Fifteen practice trials were provided for participants to familiarize themselves with the task before starting the experiment.

### 2.3 Analysis

Following Moore et al. [5] and Pilz et al. [10], reaction times of less than 150 ms were excluded as anticipatory responses and only correct responses were included in the analysis.

To estimate space- and object-based effects over time, we calculated mean reaction times (RT) for valid, invalid-same, and invalid-different trials within 51 bins of 50 ms. Starting with the cue-to-target interval of 50–100 ms, the 50-ms bin was shifted forward by 10 ms to calculate the next reaction time average (60–110 ms). This procedure was repeated across the whole range of cue-to-target intervals from 50 ms to 600 ms.

Space-based effects were calculated by subtracting the mean RTs of valid trials from the mean RTs of invalid trials for each cue-to-target interval. Alternatively, computing space-based effects by subtracting the mean RTs of valid from those of invalid-different trials (e.g., [29]) yielded nearly identical results to computing space-based effects by subtracting the mean RTs of valid from those of invalid trials. We decided to keep to the latter in the current study, as it is more common within the literature [4, 5, 10]. Object-based effects were calculated by subtracting the mean RTs of invalid-same trials from those of invalid-different trials for each cue-to-target interval. We conducted a 51 (cue-to-target interval) x 3 (valid, invalid-same, invalid-different) repeated measures ANOVA to assess overall RT differences. A Greenhouse-Geisser correction was applied when the assumption of sphericity was violated [42]. FDR-corrected t-tests were used to examine whether the cueing effects were greater than zero for each cue-to-target interval [43]. On the accuracy of responses, we followed the same data processing steps as described above. However, similar to previous studies using a similar paradigm, accuracy was high (S1A Fig in S1 File) and therefore, analyses on variabilities between individuals and the rhythmic sampling of visual space were based on RTs only.

To investigate individual variabilities in the temporal dynamics of object-based attention, we assessed space- and object-based effects for each cue-to-target interval for each participant using the percentile bootstrap method [10, 44–46]. For each participant and each condition, 999 bootstrapped data sets were constructed by randomly resampling the original data with replacement. The bootstrapped data sets contained the same number of trials in each condition as the original data. Each bootstrapped data set was analyzed like the original sample: space-based effects were calculated by subtracting the mean bootstrapped RTs of valid trials from the mean bootstrapped RTs of invalid trials. Object-based effects were calculated by subtracting the mean bootstrapped RTs of invalid-same trials from those of invalid-different trials. The 2.5 and 97.5 percentiles of the bootstrapped cueing effects were used to estimate the 95% confidence intervals for space- and object-based effects for each cue-to-target interval.

For the spectral analysis, the data from each participant were standardized for each condition to a mean of zero and a standard deviation of one. After applying a Hanning window of the same length as the data, the standardized time-series data were converted into the frequency domain using the fast Fourier transform (FFT). The FFT was carried out only on the actual time points measured (without any zero padding), yielding a lower frequency limit and frequency resolution of 1.96 Hz. The magnitudes of the FFT output were averaged across participants to estimate the average spectrum for each condition. Non-parametric statistical tests were applied to estimate the spectral significance. The non-parametric tests were computed as follows: for each participant and each condition, standardized RTs were resampled across cue-to-target intervals without replacement. The same data processing steps as for the original data (described above) were then applied to the randomized data. For each participant, this procedure was repeated 1000 times to generate reference distributions for each condition. The resulting spectra were then averaged across participants yielding 1000 average spectra for each condition. To determine the statistical significance of the frequency component, for each participant, the spectrum of each condition was compared with 1000 spectra of that condition respectively. On the group level, the average spectrum of each condition was compared with the 1000 average spectra of each condition respectively. P-values were the proportion of spectra in the reference distribution that exceeds the spectrum in the original data. P-values were corrected using the false discovery rate, accounting for multiple comparisons across the 43 participants and 26 frequencies within each condition (FDR; [43]).

## 3. Results

Accuracy was overall high for all three conditions (valid: 96%, invalid-same: 89%, invalid-different: 90%). Details can be found in the Supplemental Information and *S*1 Fig in S1 File.

### 3.1 Overall RTs

**Fig 2:**
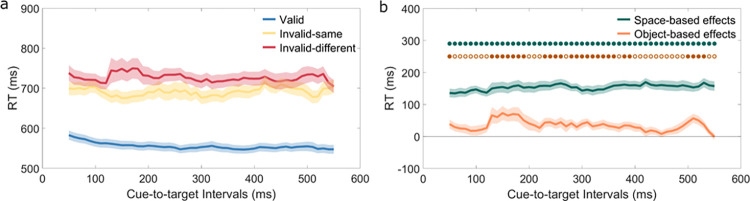
a) Mean RTs ± SEM (standard error from the mean) for valid (blue), invalid-same (yellow), and invalid-different trials (red) for all cue-to-target intervals. b) Space-based effects (green line) and object-based effects (orange line) for all cue-to-target intervals. Filled dots showed cue-to-target intervals with significant effects.

Fig 2A shows average RTs for all three conditions across cue-to-target intervals. The ANOVA showed a significant main effect of condition, *F* (1, 53) = 138.49, *p* < 0.001, η_p_^2^ = 0.77. Participants responded faster for valid trials (*M* = 555, *SD* = 7) than invalid-same trials (*M* = 691, *SD* = 9; *t* (40) = -11.89, *p* < 0.001, *Cohen’s d* = -1.86), or invalid-different trials (*M* = 726, *SD* = 9, *t* (40) = -12.67, *p* < 0.001, *Cohen’s d* = -1.98). Responses were significantly faster for invalid-same trials than invalid-different trials, *t* (40) = -5.44, *p* < 0.001, *Cohen’s d* = -0.85. The main effect of cue-to-target interval was significant, *F* (8, 341) = 2.14, *p* = 0.03, η_p_^2^ = 0.05. RTs varied between cue-to-target intervals with maximum RTs at the interval of 50 ms and minimum RTs at the interval of 300 ms. The interaction between condition and cue-to-target intervals was not significant, *F* (11,449) = 1.62, *p* = 0.09, η_p_^2^ = 0.04.

FDR-corrected t-tests showed significant space-based effects for all cue-to-target intervals: participants responded overall faster when targets appeared at cued compared to uncued locations. Object-based effects were less pronounced than space-based effects and were only significant at cue-to-target intervals between 130–190 ms, 230–260 ms, 280–360 ms, 380–390 ms, and 500–530 ms (Fig 2B).

We further compared individual standard deviations of the temporal dynamics between space- and object-based effects using paired t-tests. Inter-individual variations of the temporal dynamics for the object-based effects (Mean SD: 80 ms) were significantly greater than those for space-based effects (Mean SD: 43 ms), *t* (42) = 9.23, *p* <0.001.

To test the reliability for space- and object-based effects, we conducted a Monte Carlo splitting procedure [47]. The results of this analysis indicated a high reliability for space-based effects (0.96), whereas object-based effects exhibited relatively lower reliability (0.55). Further information and specific details can be found in Supplemental Information.

### 3.2 Bootstrapping of individual participants

**Fig 3:**
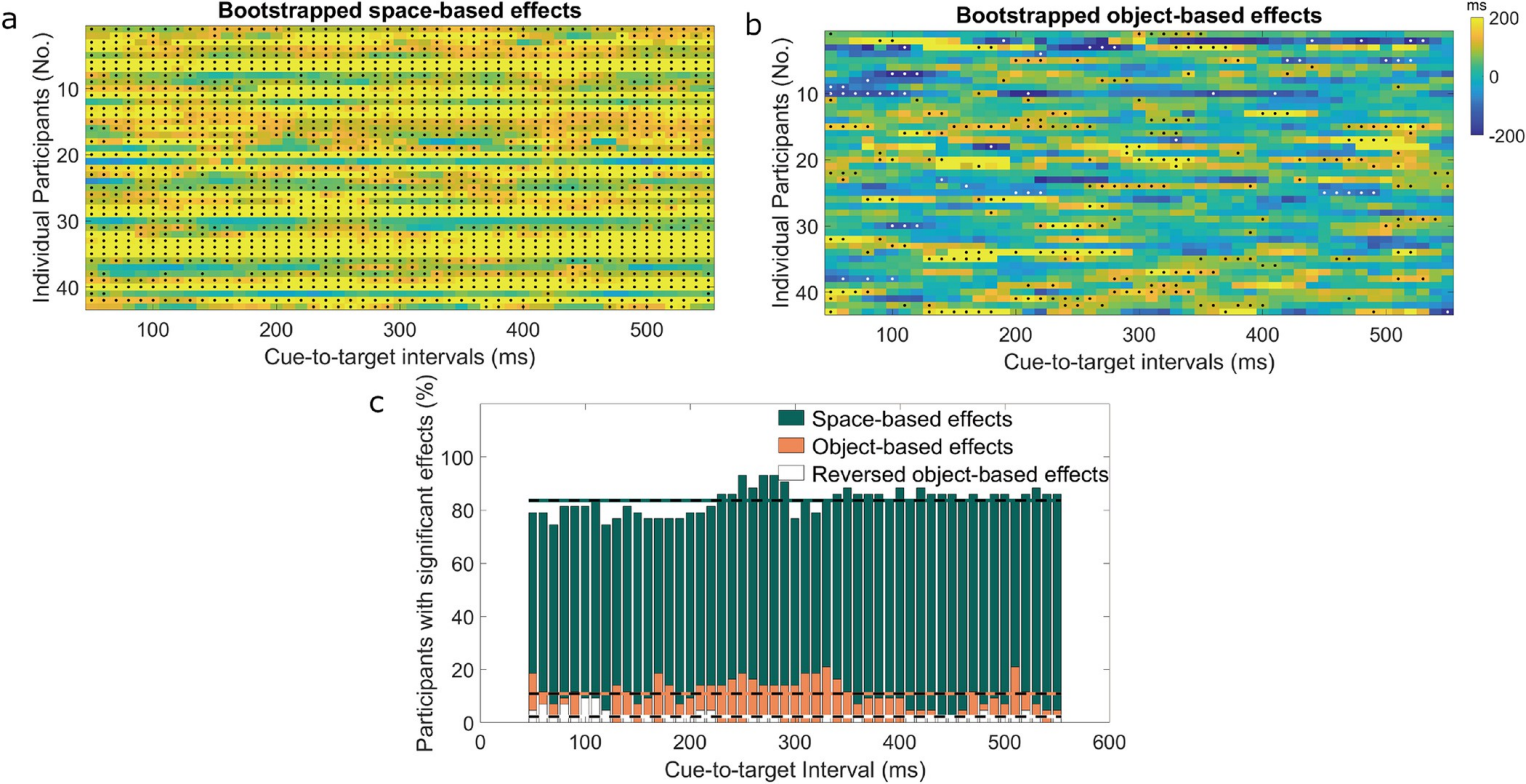
Bootstrapped space- (a) and object-based effects (b) for individual participants. Black dots indicate cue-to-target intervals with significant bootstrapped effects. White dots indicate cue-to-target intervals with significant reversed object-based effects (different-object advantage). The color scale represents the size of the object-based effect in milliseconds (ms), spanning from -200 ms (negative effects) to +200 ms (positive effects). c) The percentage of participants with significant space- (green bars), object- (orange bars), and reversed object-based effects (white bars) in reaction times for each cue-to-target interval. The average percentage of space- (84%), object- (11%) and reversed object-based effects (2%) are indicated by a green/black line, an orange/black line, and a white/black line, respectively.

Fig 3 shows bootstrapped space- and object-based effects for individual participants at each cue-to-target interval (Fig 3A and 3B) and highlights the number of participants with significant space- and object-based effects for each cue-to-target interval (Fig 3C). Within each cue-to-target interval, more than 70% of the participants showed significant space-based effects. Moreover, twenty-three participants (53%) exhibited significant space-based effects for all cue-to-target intervals, and twenty-seven participants (63%) exhibited significant space-based effects for more than 90% of all cue-to-target intervals. Thirty-eight participants (88%) showed significant space-based effects for more than half of the cue-to-target intervals. Only one participant did not exhibit any significant space-based effects.

Object-based effects were not as prevalent as space-based effects. Maximum prevalence was shown at cue-to-target intervals from 210 to 340 ms (Fig 3C). Using t-tests, we further compared the percentage of participants showing significant effects between space- and object-based effects. Space-based effects were significantly more prevalent than object-based effects, *t* (50) = 75.44, *p*< 0.001. On the level of individual participants (Fig 3B), none of the participants exhibited object-based effects for all cue-to-target intervals. Forty participants (93%) exhibited object-based effects for more than one cue-to-target interval. Thirty-three participants (77%) exhibited significant object-based effects for less than ten cue-to-target intervals. Only three participants (7%) did not exhibit any significant object-based effects. Besides, significant reversed object-based effects (different-object advantage) were found in fifteen participants (35%). *S*2 Fig in S1 File shows space- (a) and object-based effects (b) for a subset of individual participants.

In our study, the number of trials in the valid condition was greater than that in the invalid condition. To examine whether the limited number of trials available for detecting object-based effects, compared to the larger number of trials designed for detecting space-based effects, might have potentially contributed to the relatively lower occurrence of object-based effects among individual participants, we calculated bootstrapped space- and object-based effects for data in which we subsampled the number of valid trials to be equal to the number of invalid same and different trials. Results did not differ significantly from the original analysis and are highlighted in S3 Fig in S1 File. This suggests that the low prevalence of object-based effects cannot be solely attributed to the decreased number of trials.

### 3.3 Rhythmic sampling

**Fig 4:**
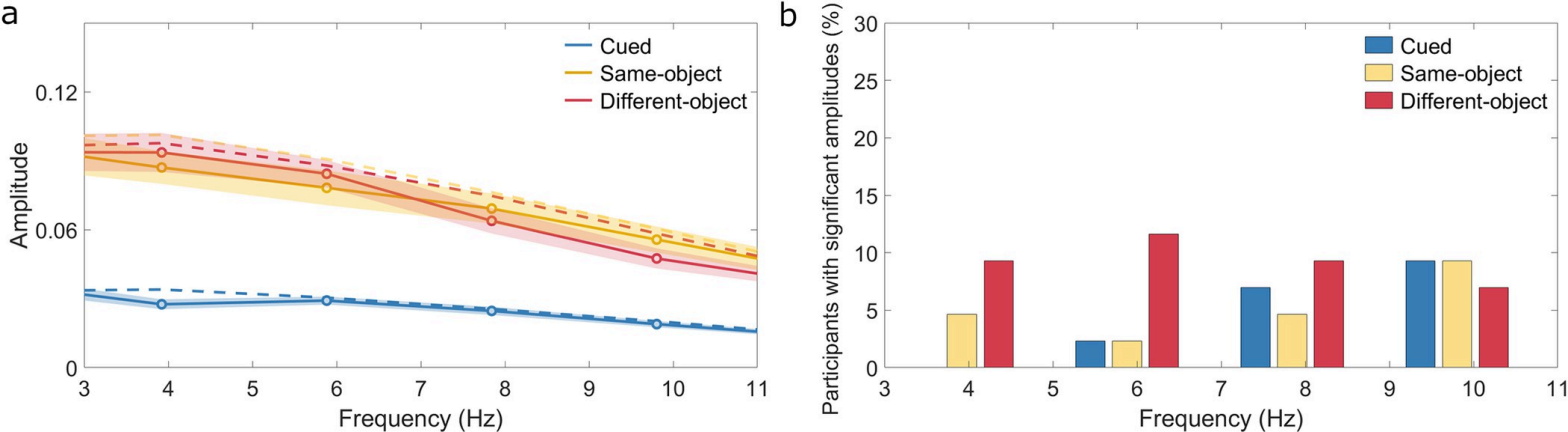
a) Average spectral amplitudes (mean± SEM) for cued location (blue), same-object location (yellow) and different-object location (red). b) Percentage of participants with significant amplitudes for cued location (blue), same- (yellow), and different-object location (red) at frequencies of 4–10 Hz.

Fig 4A shows the average amplitude spectrum for cued locations (valid trials), same-object locations (invalid-same trials), and different-object locations (invalid-different trials) across participants. Amplitudes between 4-10Hz were not significant for any condition (FDR corrected). On the individual level, few participants showed periodicity of 4, 6, 8 or 10 Hz for cued locations (N< = 4; <10%), same-object locations (N< = 4; <10%) and different-object locations (N< = 5; <12%). Fig 4B shows the percentage of participants with significant amplitudes for three locations at frequencies of 4–10 Hz.

## 4. Discussion

Recent research has shown effects of object-based attention are smaller and less prevalent than effects of space-based attention, which are only found in a small subset of participants [8, 10]. In the current study, we systematically changed the intervals between the cue and the target, varying from 50 to 600 milliseconds. We investigated whether individual variabilities in the timing of attentional selection could explain the low prevalence of object-based effects. Two different approaches were used to assess the temporal course of attentional selection: bootstrapping to investigate cue-to-target intervals with maximum object-based effects in the time domain and fast Fourier transform to investigate the rhythmic sampling of visual space in the frequency domain.

In the time domain, on the group level, we found large and stable space-based effects in reaction times across all cue-to-target intervals. However, object-based effects were small and appeared mainly at intervals between 100 and 400 ms. On the level of individual participants, most participants exhibited space-based effects in all cue-to-target intervals. In contrast, only a small number of participants showed object-based effects (<20% at each cue-to-target interval), with the highest prevalence at cue-to-target intervals between 210 and 340 ms. In the frequency domain, no rhythmic sampling at 4–10 Hz was found for all three locations: cued, same-object, and different-object locations. On the individual level, significant spectral amplitudes were only observed in a small number of participants.

The results in the time domain are consistent with previous studies indicating space-based effects are stronger and more robust than object-based effects [4, 5, 8, 10, 11, 48, 49]. These results support the findings by Pilz et al. [10], indicating that object-based effects are not as commonly observed as has previously been assumed. Similar to our study, they found object-based effects to be limited to only a small number of participants. It has been suggested that the reported object-based effects in previous studies appear to be exaggerated, likely due to inappropriate practices in data collection and analysis, as well as a lack of necessary reliability for replication [50]. The observed relatively lower reliability for object-based effects compared to space-based effects further suggests that object-based effects may not be consistently reliable.

Moreover, the current study extends upon the previous findings of Pilz et al. [10] by showing variability in the temporal dynamics of object-based effects. Our results suggest that the timing of object-based effects is not consistent across individuals. While object-based effects were observed in more than 90% of the participants, there were distinct variations in the specific time points at which these effects occurred. Participants exhibited object-based effects only at a few cue-to-target intervals. These results suggest that constant cue-to-target intervals, commonly employed in previous studies (e.g., [2, 10, 48, 51]), may have captured object-based effects for only a limited number of participants. It should be noted that considering our findings, it remains uncertain whether the temporal dynamics of object-based effects we observed are unique to specific individuals or if they remain consistent across different experiments or sessions for these individuals.

It is important to note that we only used horizontally oriented rectangles in the study, rather than randomly selecting between horizontal and vertical orientations on each trial. This decision was made due to the focus on the temporal aspects of object-based attention and the overall duration of the experiment. Previous studies have shown that attention spreads more easily along the horizontal compared to the vertical meridian [7, 40]. Using only horizontal rectangles, we could not clearly differentiate between object-based effects and those relating to an attentional advantage along the horizontal meridian. However, object-based effects have been shown to be either similar for both types of rectangles [4, 5, 8, 29, 52] or slightly enlarged for horizontal ones [7, 10, 40]. Therefore, the attentional advantage along the horizontal meridian is unable to fully explain object-based effects. In addition, if anything, we would have expected to find stronger object-based effects when using only horizontal rectangles, which is not the case.

Our findings in the frequency domain contradicted previous research suggesting the presence of rhythmic patterns underlying attentional allocation [22–25]. Previous studies have demonstrated that attention exhibits periodic sampling of visual space with frequencies ranging from 6 to 8 Hz [26, 27, 29, 32], as well as 3 to 4 Hz periodicities in the sampling of multiple locations [23, 29, 30]. Specifically, Fiebelkorn et al. [29] reported an 8 Hz periodicity for cued and same-object locations and a 4 Hz periodicity for same- and different-object locations, suggesting that attention rhythmically samples locations within and between objects. However, in our study, we did not observe significant periodic patterns at the group level, with only a few participants exhibiting periodicities.

Several experimental design differences may account for the discrepancy between the results of Fiebelkorn et al. [29] and ours. First, Fiebelkorn et al. investigated rhythmicity in attentional sampling based on detection rates (accuracy) of near-threshold targets, whereas we employed a visual discrimination task, which is in line with classical object-based paradigms [5, 8, 10], and primarily measured reaction times (due to the high accuracy in our study). It is possible that the rhythmic patterns observed in reaction times are less prominent or noticeable compared to those observed in accuracy. Second, given high cue validity, it is likely that participants on each trial moved their attention to the cued location, and moved their attention away from the cued location on invalid trials in an object-based manner, first to the same object, and then to the different object: our results show fastest responses for valid trials, but slower responses for invalid-different trials than invalid-same trials. RT differences between invalid-same and invalid-different trials were 35 ms, which corresponds to the typical serial search slope of 20–40 ms/item [53–55]. When cue validity is high, there is no strategic advantage for participants to attend to uncued locations [10, 56].

Furthermore, it is worth noting that not all studies have reported significant periodicities in attentional sampling. Some studies have failed to observe significant periodic patterns [57], or have found periodicities only at uncued locations [58, 59]. Brookshire [60] used alternative analysis techniques (AR surrogate analysis) and found that the rhythmic temporal structure in attention may not be as prevalent as previously assumed. They suggested that behavioral oscillations could be explained by autocorrelation in the time series rather than attentional periodicity. The variability observed among individuals in the frequency domain may reflect differences in the periodic patterns of attentional sampling among individuals, or it could be attributed to the inherent statistical noise within the frequency analysis itself. Therefore, further investigations are required to explore these possibilities and validate the existence of individual variations in the temporal dynamics of object-based attention.

In conclusion, the present study supports previous research indicating that object-based attention is not as prevalent as previously assumed. Further, our findings reveal individual variations in the temporal aspects of object-based attention, which may account for the infrequent occurrence of object-based effects in studies using a constant cue-to-target interval. Contrary to theories proposing rhythmic patterns in attentional allocation (e.g., [31, 61]), our findings based on reaction times in a discrimination task do not support this. However, given the relatively low reliability of object-based effects, the observed individual variations may also be influenced by statistical noise, which needs further investigation. Overall, our findings highlight the significance of taking individual variations into account when constructing and developing visual attention theories.

## Supporting information

S1 File Additional analysis. (DOCX)
