# Supplementary material for: Variability in the temporal dynamics of object-based attentional selection
Source: PLoS One. 2023 Nov 17;18(11):e0294252. doi: 10.1371/journal.pone.0294252 (PMC10656001; doi:10.1371/journal.pone.0294252)
Supplement: S1 File — (DOCX) [file pone.0294252.s001.docx]

**Additional analysis**

**Accuracy**


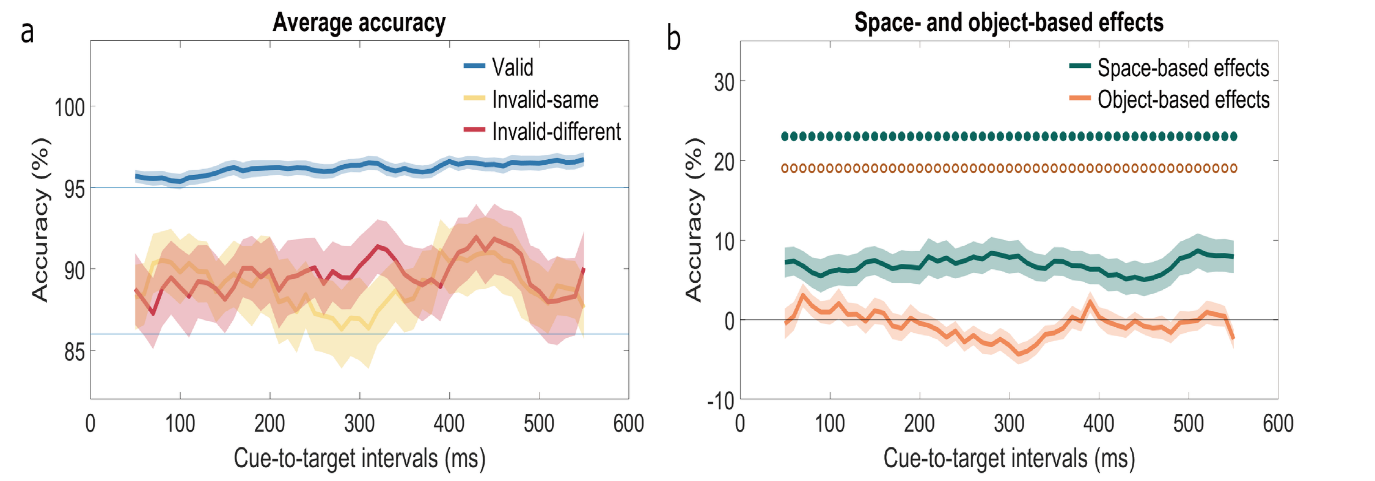
At a level of 0.05, a 51 (cue-to-target interval) x 3 (condition) repeated measures ANOVA with Greenhouse-Geisser correction showed a significant main effect of conditions, *F* (1, 47) = 13.46, *p* < 0.001, η_p_^2^ = 0.24. Accuracy was higher for valid trials (*M* = 96%, *SD* = 0.4) than accuracy for invalid trials (invalid-same trials: *M* = 89%, *SD* = 1.3; invalid-different trials: *M* = 90%, *SD* = 1.1). The main effect of cue-to-target interval (*F* (6, 263) = 1.60, *p* = 0.15, η_p_^2^ = 0.04) and the interaction (*F* (12, 516) = 1.46, *p* = 0.13, η_p_^2^ = 0.03) were not significant, indicating that the accuracy did not change across cue-to-target intervals. T-test results (FDR corrected) showed significant space-based effects across all cue-to-target intervals, whereas no significant object-based effects were found for any cue-to-target intervals (Supplemental Figure *S*1).

***Figure S1*.** a) Average accuracy (mean±1 SEM) for valid trials (blue), invalid-same trials (yellow) and invalid-different trials (red) for all cue-to-target intervals. b) Space- (green) and object-based effects (orange) in accuracy for all cue-to-target intervals. Filled dots showed cue-to-target intervals with significant effects from t-test results.


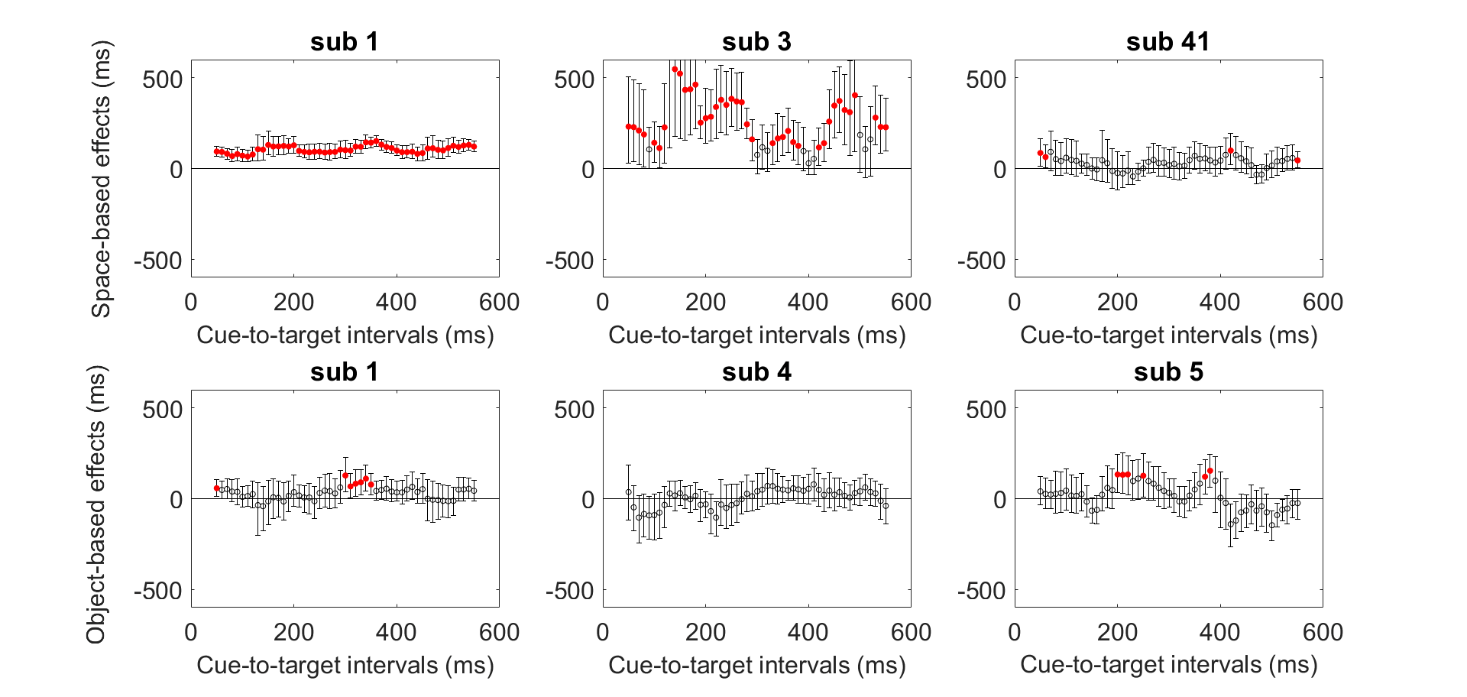
**Space- and object-based effects for individual participants**

***Figure S2*.** Examples of different patterns of space- and object-based effects across cue-to-target intervals for a selection of individual participants. The red dots showed cue-to-target intervals with significant effects.

**Bayesian statistics for the null hypothesis**

To provide an unbiased criterion with regard to the null hypothesis, we applied Bayesian statistics on the accuracy data with JASP (Wagenmakers, Love, et al., 2018; Wagenmakers, Marsman, et al., 2018). Two fitted models, one with Cue-to-target interval and one with the interaction between Cue-to-target interval and Condition, were compared separately with the null model. The results with a Bayes Factor BF_10_ <1/10 provide strong evidence in favor of the null hypothesis (Lee & Wagenmakers, 2013; Wagenmakers, Love, et al., 2018) for both the main effect of cue-to-target interval (BF_10_ = 9.57 × 10^-10^) and the cue-to-target interval x condition interaction (BF_10_ = 1.7 × 10^-9^).

**Bootstrapped results with subsampled dataset**

One concern is the unbalanced power to detect space- and object-based effects because of the far more valid trials than invalid trials in the current study. To address this concern, we first subsampled the number of valid trials to the equal number of invalid trials. We then followed the same steps as before to investigate individual variabilities in space- and object-based effects. Figures *S*3a and *S*3b showed bootstrapped space- and object-based effects for each participant and each cue-to-target interval for the subsampled data. Black dots showed cue-to-target intervals with significant effects. White dots in Figure *S*3b showed cue-to-target intervals with significant reversed object-based effects (different-object advantage). Figure *S*3c showed the percentage of participants with significant space-, object- and reversed object-based effects for each cue-to-target interval.


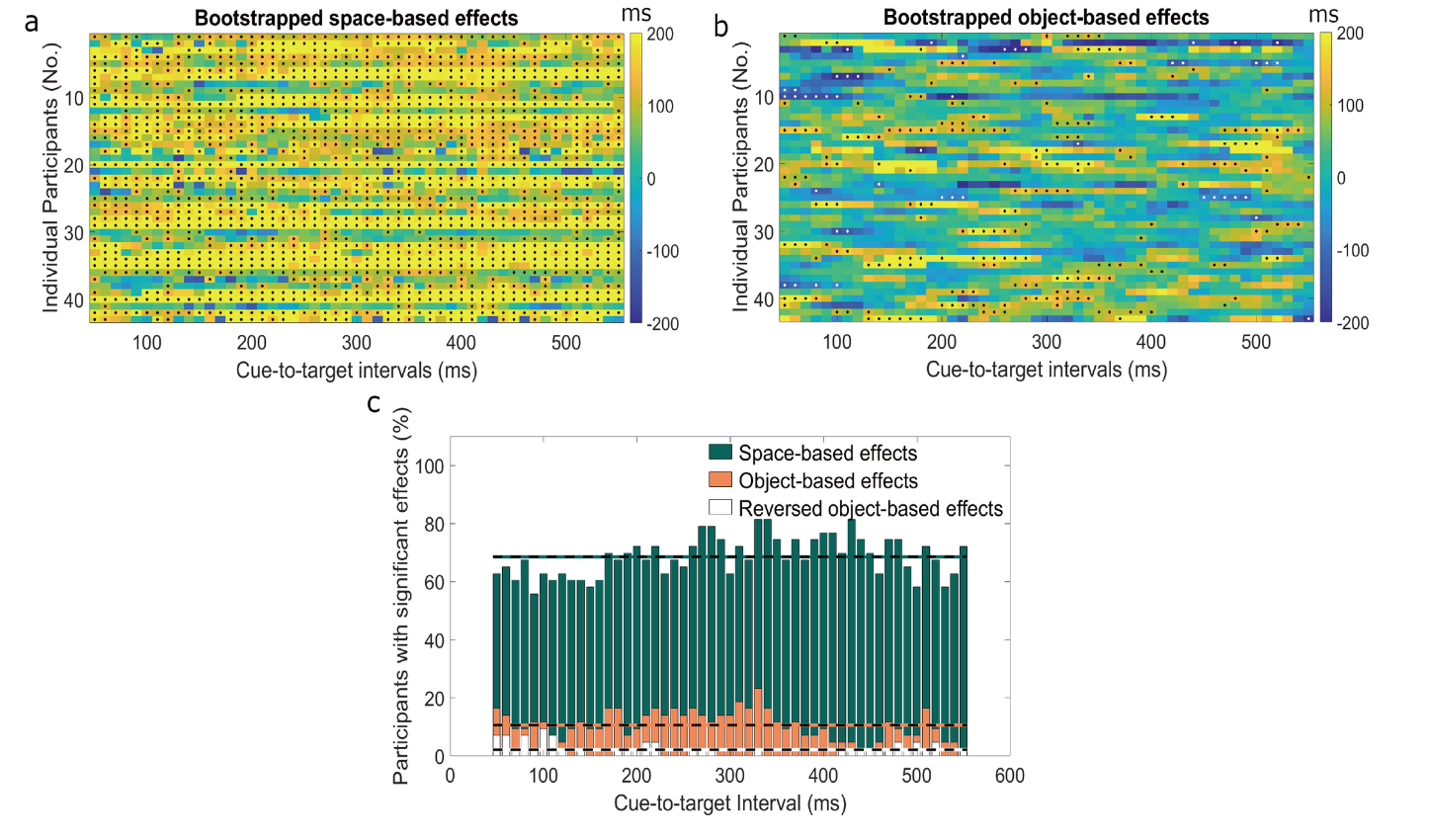
As shown in the figures, bootstrapped space-based effects (Figure *S*3a) were far more prevalent than object-based effects (Figure *S*3b). On average, 68.5% of participants showed space-based effects, whereas 10.6% of participants showed significant object-based effects and 2.1% of participants showed negative object-based effects. These results suggest that subsampling the number of valid trials did not affect the observed pattern of space- and object-based effects to a great extent.

***Figure S3*.** a) Bootstrapped space-based effects in reaction time for individual participants for each cue-to-target interval. b) Bootstrapped object-based effects in reaction time for individual participants for each cue-to-target interval. Black dots indicate cue-to-target intervals with significant bootstrapped effects. White dots showed cue-to-target intervals with significant reversed object-based effects (different-object advantage). c) The percentage of participants with significant space- (green bar), object- (orange bar), and reversed object-based effects (white bars) in reaction times for each cue-to-target interval. Average percentage of space- (68.5%) and object- (10.6%) and reversed object-based effects (2.1%) were indicated by green/black line, orange/black line and white/black line respectively.

**Split-half reliability analysis**

To test the reliability for space- and object-based effects, we conducted a Monte Carlo splitting procedure (Williams & Kaufmann, 2012), as it being a more robust method estimating the reliability (Pronk et al., 2022). The procedure is as follows: we first constructed two parts of data that have the same length as the original data by randomly sampling with replacement. Next, we calculated space- and object-based effects for each dataset. This process was repeated 5000 times (Parsons et al., 2019), and the Spearmen correlation coefficients were calculated. After that, we calculated the average coefficients for the space-based and object-based effects by averaging them across the 5000 iterations.

This analysis revealed a high reliability of space-based effects (mean: 0.9595), but a relatively low reliability of object-based effects (mean: 0.5473, from 0.1542 to 0.8502), falling below the acceptable threshold of 0.7 according to conservative standards (Nunnally ,1978). The results confirm our assumption that space-based effects are robust, whereas object-based effects are less reliable.
